# Supplementary material for: Physiological roles of endocytosis and presynaptic scaffold in vesicle replenishment at fast and slow central synapses
Source: eLife. 2024 Jun 3;12:RP90497. doi: 10.7554/eLife.90497 (PMC11147502; doi:10.7554/eLife.90497)
Supplement: Supplementary file 1. [file elife-90497-supp1.docx]

| Stimulation(s) | Parameters | | Control | Dynasore | Pitstop-2 | Dyn-1 PRD | ML141 | Lat-B |
| --- | --- | --- | --- | --- | --- | --- | --- | --- |
| 5 ms | ΔCm (fF) | Mean (± SEM) | 295 (± 27) | 280 (± 32) | 236 (± 30) | 249 (± 19) | 260 (± 47) | 343 (± 45) |
|  |  | *p*-value | - | 0.73 | 0.18 | 0.20 | 0.54 | 0.40 |
|  |  |  |  |  |  |  |  |  |
|  | ICa (nA) | Mean (± SEM) | 1.24 (± 0.24) | 1.05 (± 0.13) | 1.05 (± 0.21) | 0.87 ± (0.11) | 0.88 (± 0.29) | 0.98 (± 0.2) |
|  |  | *p-*value | - | 0.51 | 0.58 | 0.21 | 0.36 | 0.43 |
|  |  |  |  |  |  |  |  |  |
|  | Endo rate (fF/s) | Mean (± SEM) | 28.8 (± 3.7) | 10.3 (± 2.8) | 10.1 (± 3.4) | 2.3 (± 2.2) | 23.0 (± 5.8) | 25.6 (± 5.2) |
|  |  | *p*-value | - | 0.004 | 0.006 | < 0.001 | 0.44 | 0.65 |
|  |  |  |  |  |  |  |  |  |
|  |  | (n) | 5 | 5 | 5 | 5 | 6 | 6 |
|  |  |  |  |  |  |  |  |  |
| 20 ms x 15_1 Hz | Cumulative ΔCm (pF) | Mean (± SEM) | 2.41 (± 0.16) | 1.25 (± 0.08) | 1.67 (± 0.08) | 1.7 (± 0.21) | 1.87 (± 0.1) | 2.05 (± 0.3) |
|  |  | *p*-value | - | 0.0007 | 0.004 | 0.02 | 0.02 | 0.28 |
|  |  |  |  |  |  |  |  |  |
|  | Endo rate (fF/s) | Mean (± SEM) | 251 (± 28.3) | 121 (± 19.1) | 133 (± 15.6) | 132 (± 9.8) | 214 (± 19.1) | 233 (± 47.6) |
|  | (Last four points average) | *p*-value | - | 0.004 | 0.007 | 0.001 | 0.3 | 0.7 |
|  |  |  |  |  |  |  |  |  |
|  |  | (n) | 6 | 6 | 5 | 7 | 5 | 4 |
|  |  |  |  |  |  |  |  |  |
| 20 ms x 10_10 Hz | Cumulative ΔCm (pF) | Mean (± SEM) | 1.67 (± 0.15) | 1.03 (± 0.09) | 1.0 (± 0.12) | 1.03 (± 0.1) | 1.31 (± 0.11) | 1.42 (± 0.3) |
|  |  | *p*-value | - | 0.008 | 0.009 | 0.007 | 0.09 | 0.49 |
|  |  |  |  |  |  |  |  |  |
|  | Endo rate (fF/s) | Mean (± SEM) | 346 (± 40.1) | 204 (± 25.3) | 131 (± 24.6) | 161 (± 27.3) | 242 (± 21.5) | 350 (± 33.3) |
|  |  | *p*-value | - | 0.017 | 0.004 | 0.003 | 0.08 | 0.95 |
|  |  |  |  |  |  |  |  |  |
|  |  | (n) | 6 | 4 | 4 | 6 | 4 | 5 |
|  |  |  |  |  |  |  |  |  |
| Figure(s): | |  | 1, 3 and Fig1-Fig Supp1 | 1 | | Fig1-Fig Supp1 | 3 | |
|  |  |  |  |  |  |  |  |  |
| Endocytosis rate was measured from following time frame(s) | | | | | | | | |
|  | 1) 5 ms: (0.45 - 5.45) s after stimulation | | | | | | |  |
|  | 2) 20 ms x 15_1 Hz: (0.45 - 0.95) s after every stimulation | | | |  |  |  |  |
|  | 3) 20 ms x 10_10 Hz: (0.45 - 1.45) s after last stimulation | | | | | | |  |
