# Supplementary material for: Physiological roles of endocytosis and presynaptic scaffold in vesicle replenishment at fast and slow central synapses
Source: eLife. 2024 Jun 3;12:RP90497. doi: 10.7554/eLife.90497 (PMC11147502; doi:10.7554/eLife.90497)
Supplement: Supplementary file 2. [file elife-90497-supp2.docx]

| Frequency | Conditions/parameters | | | Tau fast (s) | Tau slow (s) | Tau mean (s) | Af/As | (n) | Figure (s) |
| --- | --- | --- | --- | --- | --- | --- | --- | --- | --- |
| 10 Hz | I∆t/I1st | Control | Mean (± SEM) | - | - | 2.3 (± 0.4) | - | 9 |  |
|  |  | Dynasore | Mean (± SEM) | - | - | 1.7 (± 0.2) | - | 8 |  |
|  |  |  | *p*-value |  |  | 0.2 |  |  |  |
|  |  | Pitstop-2 | Mean (± SEM) | - | - | 1.9 (± 0.3) | - | 6 |  |
|  |  |  | *p*-value |  |  | 0.5 |  |  |  |
|  |  | ML141 | Mean (± SEM) | - | - | 2.6 (± 0.2) | - | 8 |  |
|  |  |  | *p*-value |  |  | 0.61 |  |  |  |
|  |  | Lat-B | Mean (± SEM) | - | - | 3.7 (± 0.9) | - | 6 |  |
|  |  |  | *p*-value |  |  | 0.14 |  |  |  |
|  |  |  |  |  |  |  |  |  |  |
|  | (I∆t - Iss)/(I1st - Iss) | Control | Mean (± SEM) | - | - | 2.2 (± 0.4) | - | 9 | 2, 4 |
|  | Normalized at Iss | Dynasore | Mean (± SEM) | - | - | 1.8 (± 0.3) | - | 8 | 2 |
|  |  |  | *p*-value |  |  | 0.32 |  |  |  |
|  |  | Pitstop-2 | Mean (± SEM) | - | - | 2.1 (± 0.24) | - | 6 | 2 |
|  |  |  | *p*-value |  |  | 0.9 |  |  |  |
|  |  | ML141 | Mean (± SEM) | - | - | 3.0 (± 0.5) | - | 8 | 4 |
|  |  |  | *p*-value |  |  | 0.21 |  |  |  |
|  |  | Lat-B | Mean (± SEM) | - | - | 3.4 (± 0.6) | - | 6 | 4 |
|  |  |  | *p*-value |  |  | 0.16 |  |  |  |
|  |  |  |  |  |  |  |  |  |  |
| 100 Hz | I∆t/I1st | Control | Mean (± SEM) | 0.21 (± 0.06) | 3.5 (± 0.5) | 1.9 (± 0.4) | 1.4 (± 0.3) | 8 |  |
|  |  | Dynasore | Mean (± SEM) | 0.069 (± 0.01) | 2.0 (± 0.3) | 0.93 (± 0.06) | 1.3 (± 0.2) | 9 |  |
|  |  |  | *p*-value | 0.02 | 0.008 | 0.01 | 0.7 |  |  |
|  |  | Pitstop-2 | Mean (± SEM) | 0.06 (± 0.014) | 1.8 (± 0.24) | 0.8 (± 0.1) | 1.5 (± 0.4) | 6 |  |
|  |  |  | *p*-value | 0.034 | 0.008 | 0.02 | 0.8 |  |  |
|  |  | ML141 | Mean (± SEM) | 0.14 (± 0.03) | 2.3 (± 0.2) | 1.3 (± 0.2) | 1.1 (± 0.2) | 8 |  |
|  |  |  | *p*-value | 0.23 | 0.04 | 0.17 | 0.45 |  |  |
|  |  | Lat-B | Mean (± SEM) | 0.07 (± 0.014) | 2.6 (± 0.5) | 1.43 (± 0.2) | 0.9 (± 0.15) | 8 |  |
|  |  |  | *p*-value | 0.04 | 0.2 | 0.3 | 0.2 |  |  |
|  |  |  |  |  |  |  |  |  |  |
|  | (I∆t - Iss)/(I1st - Iss) | Control | Mean (± SEM) | 0.18 (± 0.05) | 3.2 (± 0.5) | 1.8 (± 0.4) | 1.5 (± 0.4) | 8 | 2, 4 |
|  | Normalized at Iss | Dynasore | Mean (± SEM) | 0.05 (± 0.009) | 1.5 (± 0.2) | 0.78 (± 0.09) | 0.9 (± 0.12) | 9 | 2 |
|  |  |  | *p*-value | 0.008 | 0.003 | 0.02 | 0.24 |  |  |
|  |  | Pitstop-2 | Mean (± SEM) | 0.04 (± 0.014) | 1.3 (± 0.3) | 0.53 (± 0.08) | 1.6 (± 0.4) | 6 | 2 |
|  |  |  | *p*-value | 0.02 | 0.008 | 0.02 | 0.8 |  |  |
|  |  | ML141 | Mean (± SEM) | 0.13 (± 0.024) | 2.3 (± 0.3) | 1.3 (± 0.2) | 1.1 (± 0.2) | 8 | 4 |
|  |  |  | *p*-value | 0.4 | 0.15 | 0.2 | 0.4 |  |  |
|  |  | Lat-B | Mean (± SEM) | 0.07 (± 0.02) | 2.5 (± 0.4) | 1.6 (± 0.3) | 0.73 (± 0.23) | 8 | 4 |
|  |  |  | *p*-value | 0.05 | 0.4 | 0.63 | 0.42 |  |  |
| Time constants were obtained by fitting recovery points of individual cells and taking average across cells. | | | | | | | | | |
| number of cells (n) at ∆t | | | | | | | | | |
| Frequency | ∆t (s) -> | 0.02 | 0.1 | 0.3 | 1 | 3 | 8 | 12 | 20 |
| 10 Hz | Control | - | - | 11 | 10 | 10 | 10 | 9 | 8 |
|  | Dynasore | - | - | 8 | 8 | 8 | 8 | 8 | 6 |
|  | Pitstop-2 | - | - | 7 | 7 | 7 | 7 | 7 | 7 |
|  | ML141 | - | - | 9 | 8 | 8 | 8 | 8 | 8 |
|  | Lat-B | - | - | 7 | 7 | 7 | 7 | 7 | 7 |
|  |  |  |  |  |  |  |  |  |  |
| 100 Hz | Control | 11 | 10 | 10 | 9 | 10 | 9 | 5 | 4 |
|  | Dynasore | 10 | 10 | 9 | 9 | 9 | 9 | 7 | 7 |
|  | Pitstop-2 | 6 | 7 | 7 | 7 | 7 | 7 | 7 | 7 |
|  | ML141 | 10 | 8 | 8 | 8 | 8 | 8 | 8 | 8 |
|  | Lat-B | 8 | 8 | 8 | 8 | 8 | 8 | 8 | 8 |
| Recovery curves in Figure 2, 4, and Figure 2 - figure supplement 3 were obtained by fitting the average recovery points at every ∆t time. | | | | | | | | | |
| Individual cells that recovered completely within 8 s or 12 s, without having further data points (at 12 s and/or 20 s), were extrapolated for fitting. | | | | | | | | | |
